# Nuclear and Mitochondrial Genome, Epigenome and Gut Microbiome: Emerging Molecular Biomarkers for Parkinson’s Disease

**DOI:** 10.3390/ijms22189839

**Published:** 2021-09-11

**Authors:** Gleyce Fonseca Cabral, Ana Paula Schaan, Giovanna C. Cavalcante, Camille Sena-dos-Santos, Tatiane Piedade de Souza, Natacha M. Souza Port’s, Jhully Azevedo dos Santos Pinheiro, Ândrea Ribeiro-dos-Santos, Amanda F. Vidal

**Affiliations:** 1 Laboratório de Genética Humana e Médica, Universidade Federal do Pará, R. Augusto Correa, Belém 66075-110, Brazil; cabralffg@gmail.com (G.F.C.); apschaan@gmail.com (A.P.S.); giovannaccavalcante@gmail.com (G.C.C.); camillebiologia@gmail.com (C.S.-d.-S.); tati_souz14@outlook.com (T.P.d.S.); jhully.asp@gmail.com (J.A.d.S.P.); 2 Laboratório de Neurofarmacologia Molecular, Universidade de São Paulo, São Paulo 05508-000, Brazil; natachamsports@gmail.com; 3 Núcleo de Pesquisas em Oncologia, Universidade Federal do Pará–R. dos Mundurucus, Belém 66073-000, Brazil; 4 Programa de Pós-Graduação em Genética e Biologia Molecular, Universidade Federal do Pará, R. Augusto Correa, Belém 66075-110, Brazil; 5 ITVDS—Instituto Tecnológico Vale Desenvolvimento Sustentável–R. Boaventura da Silva, Belém 66055-090, Brazil

**Keywords:** Parkinson’s disease, neurodegeneration, genetics, non-coding RNAs, microbiome, mitochondria, epigenetics, biomarkers, precision medicine

## Abstract

Background: Parkinson’s disease (PD) is currently the second most common neurodegenerative disorder, burdening about 10 million elderly individuals worldwide. The multifactorial nature of PD poses a difficult obstacle for understanding the mechanisms involved in its onset and progression. Currently, diagnosis depends on the appearance of clinical signs, some of which are shared among various neurologic disorders, hindering early diagnosis. There are no effective tools to prevent PD onset, detect the disease in early stages or accurately report the risk of disease progression. Hence, there is an increasing demand for biomarkers that may identify disease onset and progression, as treatment-based medicine may not be the best approach for PD. Over the last few decades, the search for molecular markers to predict susceptibility, aid in accurate diagnosis and evaluate the progress of PD have intensified, but strategies aimed to improve individualized patient care have not yet been established. Conclusions: Genomic variation, regulation by epigenomic mechanisms, as well as the influence of the host gut microbiome seem to have a crucial role in the onset and progress of PD, thus are considered potential biomarkers. As such, the human nuclear and mitochondrial genome, epigenome, and the host gut microbiome might be the key elements to the rise of personalized medicine for PD patients.

## 1. Introduction

As life expectancy rises as a result of technological advances, humanity faces an increased burden of aging diseases, such as cancer, diabetes, cardiovascular and neurodegenerative disorders. Degenerative diseases affecting the nervous system are recognized as major causes of death and disabilities among the elderly population worldwide [1]. However, the molecular mechanisms engaged in the onset and progression of neurodegenerative diseases remain elusive. A complete understanding of the molecular biology of neurodegeneration will benefit the search for biomarkers to be employed in strategies for disease detection and patient management, as seen with the efforts being made towards cancer research [2,3].

Among the most common neurodegenerative disorders, Parkinson’s Disease (PD) has gained a leading position, preceded only by Alzheimer’s Disease [1,4,5], affecting 1% of individuals above 60 years old and 3% of the elderly above 80 years old, and may also rarely affect individuals under 50 years old (early PD and juvenile PD) [4,6,7]. According to recent reports, there may be about 10 million individuals living with PD worldwide, a number that is predicted to multiply three-fold in the next few decades, as the elderly population grows.

Despite all the advances, the diagnosis of PD is based mainly on the observation of classic parkinsonism symptoms, such as muscle rigidness, dyskinesia, and tremor leading to postural imbalance and the investigation of family history of PD [8]. Individuals with PD also present other non-motor symptoms—most of which appear after 40–50% of neuronal loss, including the development of cognitive impairment and Parkinson-related dementia [9,10,11]. However, most PD patients are diagnosed in late stages, both because of the lack of tools for the evaluation of disease progress risk and the difficulty in differentiating PD from other neurological disorders, since many symptoms of PD overlap with clinical manifestations of other diseases, such as Essential Tremor, Multiple Sclerosis, and Alzheimer’s Disease [12].

Currently, the lack of molecular markers to predict susceptibility, accurate diagnosis, and evaluate the progress of PD continues to hinder the establishment of precision medicine strategies. Moreover, it is essential that we consider the findings of multi-omics approaches, which reveal molecular aspects of PD from multiple perspectives and may lead to the establishment of genetic and epigenetic and other circulating markers, which are less invasive, to be used for accurate diagnosis and clinical management of the disease. Here, we discuss findings concerning the identification and validation of potential genetic, epigenetic and microbial biomarkers to enlighten the state-of-the-art in PD molecular biomarker research.

## 2. Parkinson’s Disease

Since James Parkinson’s first medical description in the 19th century, PD has been defined as a neurodegenerative disorder caused by progressive and irreversible degeneration of dopaminergic neurons of the substantia nigra (SN) pars compacta (SNpc) [9,13], although Parkinsonism descriptions can be found in earlier fragments [14,15]. PD is the principal cause of Parkinsonism, which describes a syndrome defined by muscular rigidity, resting tremor and bradykinesia [16]. Regarding the dopaminergic neuron degeneration in SNpc, the main feature that characterizes PD pathology is a progressive reduction in levels of dopamine (60–80%), a neurotransmitter involved in various brain functions, such as learning, memory, mood and sleep regulation [9,17,18].

This degenerative loss of neurons in substantia nigra occurs most profoundly in the lateral–ventral tier, which contains neurons that project to the dorsal putamen of the striatum. Therefore, progressive loss of neurons in this brain area explains the major clinical symptoms [19]. Another relevant hallmark in PD is Lewy pathology. The Lewy body is a neuronal inclusion not only found in substantia nigra but also in others brain regions in PD, mainly composed of altered neurofilaments that aggregation [20]. α-synuclein (α-syn) was identified as the main aberrantly folded protein that aggregates to form inclusions, called Lewy bodies, in PD [21].

PD is considered a heterogeneous disease that can progress slowly or quickly, depending on several factors, many of which are still not well understood [22]. It is commonly divided into (a) monogenic or familial PD, which the main cause is related to specific mutations present in key genes within members of the same family; and (b) idiopathic or sporadic PD, found in 85–90% of cases and whose etiology is still unknown [23]. In addition, α-syn is encoded by the *SNCA* gene, in which the first causal missense mutation was identified to be related to the monogenic form of this disease [24].

Therapeutic strategies for many of the disabling motor features are insufficient due to poor response rates to dopaminergic therapies or the development of long-term complications after its use, likely caused by the late appearance of clinical symptoms, in which there is severe damage in SNpc [25]. Thus, personalized and more efficient therapeutic strategies must be developed.

**Figure 1:**
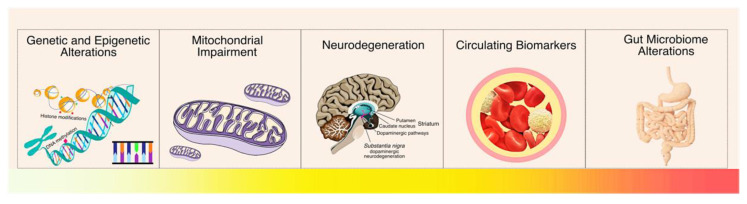
Molecular factors that may be involved in Parkinson’s Disease onset and progression. There are various points of view in PD biomarker research, thus, there is a lot to be understood from the earliest molecular alterations until de development of the firsts symptoms. In this review, we decided to discuss these multi-omics factors to highlight how they can be applied for preventive measures.

Currently, there are no effective tools to prevent PD onset, as there is no method of detecting the disease in its early stages or that can accurately inform the risk of disease progression. Consequently, further studies are needed to determine molecular factors that can be used as disease-specific biomarkers in preventive medicine (Figure 1). These can assist in the detection of PD before the onset of motor symptoms or in cases where the symptoms presented by the patient are insufficient for an accurate diagnosis. In addition to allowing an increase in the accuracy in the diagnosis process, these biomarkers could also differentiate PD from other forms of parkinsonism and other neurodegenerative diseases [6]. With this, many studies have explored the role of different genetic and epigenetic alterations, as well as alterations in the gut microbiome that may be involved in PD onset and progress, aiming to identify PD biomarkers and novel therapeutic targets.

## 3. Genetic Alterations in Parkinson’s Disease

The characterization of the genetic architecture of PD is essential for understanding the cascade of events that leads to PD onset and to find genetic biomarkers to identify individuals or populations at risk of developing PD and patient prognosis. Hence, over the last few decades, various studies investigated the relationship between monogenic and/or idiopathic PD and gene variants (Table 1). Several genes have been associated with monogenic, sporadic, and both forms of PD. Among these, variants in the *SNCA* gene, which encodes α-syn, are the main genetic factor associated with PD in both etiologies [25,26]. Under pathological conditions, α-syn cannot fulfill its physiological functions, plaques of α-syn aggregates are formed, which may be toxic [25,27]. In addition, α-syn is an inhibitor of tyrosine hydroxylase (TH), an essential enzyme for dopamine production [28,29,30], and it may also interact with dopamine transporters (DAT), affecting the dopaminergic synapse [31]. Moreover, α-syn may undergo exocytosis and be absorbed by nearby astrocytes and microglia, forming more aggregates, which stimulates the release of pro-inflammatory cytokines and chemokines—such as interleukins, Tumor Necrosis Factor α (TNF-α), and interferon-γ (IFN-γ) [32].

Further, α-syn aggregation increases the production of reactive oxygen (ROS) and nitrogen species (RNS) [32,57,58]. Under healthy conditions, ROS and RNS molecules are readily neutralized. However, in neurons affected by advanced neurodegenerative processes, the decline in production and functionality of ROS and RNS neutralizing enzymes—such as Superoxide Dismutase (SOD), Catalase (CAT), Glutathione (GSH), and Glutathione Peroxidase (GPx), intensifies the damage caused by oxidative stress [59,60]. This leads to the development of microenvironmental alterations that affect the entire neuronal circuitry and stimulates immune response through the activation of Toll-like receptors (TLRs) present in the brain microglia [61]. In turn, this initiates a cascade of events that culminates in the activation of NFκb, which mediates the expression of genes related to the inflammatory response, like cytokines, IFN-γ, TNF-α, complement proteins, and other pro-inflammatory mediators that reactivate TLRs, creating a state of chronic inflammation [57,62,63].

Moreover, the literature also shows that α-syn is involved in several mitochondrial functions such as cell respiration, mitochondrial membrane potential (MMP) modulation, and mitochondrial integrity [64], hence, gene variants may also lead to mitochondrial impairment. Additionally, *SNCA* may induce ER stress and intracellular release of Ca^2+^, leading to mitochondrial dysfunctions, which are the main cause of increased production of ROS and RNS, and activation of pro-apoptotic pathways [65,66,67].

In fact, several variants in nuclear genes essential for the pathogenesis of PD can lead to mitochondrial dysfunctions. For example, the *PARK2* and *PARK6* genes (which encode parkin and PINK1 proteins, respectively) are essential for mitophagy—a process in which damaged mitochondria are selectively degraded, crucial for functional mitochondria maintenance in senescent cells, such as neurons [68].

The *LRRK2* gene, the most frequent cause of late-onset autosomal dominant and sporadic PD [69], contributes to the recognition of damaged mitochondria by promoting the inhibition of mitochondrial motility [70], also being important for mitochondrial fusion/fission processes [71]. The DJ-1 protein, encoded by the *PARK7* gene, is associated with the recognition and neutralization of ROS and modulates the MMP, being important for the regulation of calcium levels and the stabilization of anti-apoptotic protein Bcl-X [64,72,73]. Variants in the glucocerebrosidase gene (*GBA*) were also found in both familial and idiopathic cases of PD and there is evidence that it may be involved in early-onset PD [39,74,75].

The activation of transposable elements (TE) in brain cells has also been a topic of interest in PD research, since retrotransposons such as *LINE-1*. These ancient viral particles (which were inserted in the human genome throughout the evolution) remain mostly inactive in our genome due to their potential to cause impairments in functional genes, however, they can be reactivated in the aging brain. In fact, it has been suggested that TE insertions are more common in brain cells [76,77,78]. Their activation leads to cellular mosaicism which is important for neuronal evolution [79]. Moreover, it is suggested that TE retrotransposition may also regulate gene expression in Neuronal Precursor Cells (NPCs) and differentiated neurons [78,80,81]. Despite that, TE insertions can also cause DNA damage and gene expression disruption, and their transcription may trigger microglial antiviral response, activating inflammatory pathways [76,82].

Furthermore, TE recombination events are associated with early-onset PD. Bravo et al., (2018) reported five structural variations in the *PRKN* gene, which is the most frequently mutated gene in this condition, including deletions and exon rearrangements, all of which were caused by recombinatory events triggered by TE reactivation [83]. It was also reported that pathogenic Tau protein leads to a decrease in piRNA-mediated regulation, which increases TE retrotransposition in PD neurons, triggering oxidative stress and cell death [84,85]. Although the mechanisms of TE reactivation in the brain remain elusive, the reported piRNA-mediated regulation of TEs in mammalian brain cells may be an important mechanism of neuroprotection and their deregulation may be involved in the development of neurodegenerative disorders as PD [86].

The investigation of pathogenic variants in PD-related genes is important to characterize the disease at a population level and better understand disease progression and clinical presentations in diverse patient groups, as what has been done for other diseases [87]. Nonetheless, most known variants were identified in populations of Europe, South and East Asia, and North America. For instance, recent studies reported pathogenic variants in *PARK2* and *LRRK2* in Spanish PD patients, and rare variants in *ATP13A2* and *GIGYF2* that may contribute to PD risk on a population scale [88]. Moreover, Wu et al., (2021) [89] showed an association between the p.V16A variant in superoxide dismutase gene (*SOD2*) and PD in Han Chinese individuals, while a large-scale investigation in the UK revealed copy number variants in the *SNCA* gene region in individuals without phenotypic PD manifestation [89,90]. Furthermore, a study performed by the *International Parkinson’s Disease Genomics Consortium* showed 24 novel risk loci for PD in the European population [91]; and 17 risk loci for PD in *ITPKB* and *ZNF184* were identified in a study with East Asians [92]. On the other hand, the contribution of Amerindian and/or African Ancestry to the development of PD in populations of Central and South America remains unknown, mostly due to insufficient data. Thus, it is important to highlight the importance of these studies since they may enlighten individual and populational variations in the course of the disease.

Although it is not completely understood, the genetic aspects of PD have been widely explored, in both nuclear and mitochondrial genomics. Recent studies point to the influence of epigenetic aspects in PD onset, as well as how host–microbiome interactions may influence PD progression. These findings are a valuable source of new biomarkers and therapeutic targets that may not only help us understand the mechanisms involved in neurodegeneration, but also provide improvements in clinical practice and patient management through the comprehension of differences and particularities of each patient and population. As highlighted above, a lot of knowledge was elucidated using brain tissue biomarkers. Currently, though, it is important to convert this knowledge into less invasive biomarkers, as circulating molecules altered in tissue and biofluids, which could be useful in the patient management in precision medicine approaches.

## 4. Epigenetic Alterations in Parkinson’s Disease

Advances in sequencing technologies allowed the exploration of the role of alterations not related to the DNA sequence, but associated with mechanisms of gene regulation. The main mechanisms involved in this process are DNA methylation, histone modifications and the regulation performed by non-coding RNAs (ncRNAs). Their modulation is involved in processes such as modulation of cell homeostasis and the onset of human diseases [93]. Among the various mechanisms of epigenetic regulation, the role of ncRNAs has been largely explored in different organs and tissues, including the brain. However, the epigenetic modulation of neurons remains elusive and a controversial issue.

We still do not fully understand how epigenetic modulators are involved in neuron activity, survival and death. Nonetheless, there is evidence that they may be important in healthy conditions and may contribute to the onset and progression of neurodegenerative disorders. These mechanisms may be an important source of PD biomarkers and therapeutic targets, since the epigenetic regulators can be modulated in patients to return to a healthy condition [94,95,96]. Below, we discuss some of the main findings regarding the role of ncRNAs in neurons and evidence that shows how these molecules may be involved in neurodegeneration and PD development, focusing mainly on microRNAs (miRNAs), Piwi-interacting RNAs (piRNAs) and Circular RNAs (circRNAs).

### 4.1. Chromatin Remodeling Mechanisms

**Figure 2:**
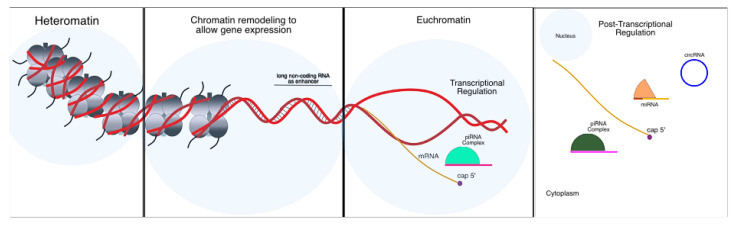
Mechanisms of gene expression regulation. Gene expression is regulated by a network which includes chromatin modulators, with the addition of molecules such as acetyl and/or methyl in histones and the methylation of DNA strands. Additionally, ncRNAs participated in several steps both in transcriptional and post-transcriptional levels. Within the nucleus, ncRNAs may play the role of enhancers, transcription factors or as gene silencing tools. In cytoplasm, they may inhibit RNA translation or modulate the action of other ncRNAs and be used as a sponge of RBP, inducing mRNA degradation.

The mechanisms of DNA methylation and histone modifications regulate the accessibility of DNA strands to transcription factors, enhancers and RNA polymerase, thus interfering directly in the transcription process (Figure 2). The involvement of DNA methylation and histone modifications in PD has been recently reported, suggesting a mechanism of genome reprogramming. Young et al., (2019) reported the alteration of DNA methylation profile in patients with PD, mainly located not only in dopaminergic neurons but also in regions such as the Cingulate gyrus and, especially, the dorsal motor nucleus of the vagus nerve, which is one of the first areas affected in early PD stages [97]. Thus, it is possible that the deregulation in DNA methylation patterns may explain the gastrointestinal symptoms related to the pre-diagnostic stages.

Alterations in DNA methylation profile were also found in blood and saliva samples of patients living with PD, mainly affecting regions harboring genes related to the immune response, leading to an alteration in blood cells composition [98]. More recently, Wang et al., (2019) found 85 hypomethylated and hypermethylated genes in blood samples of patients with PD, suggesting PD-associated blood biomarkers [99]. It is important to emphasize studies investigating PD biomarkers present in blood and other biofluids, since the sample collection of these fluids is less invasive, therefore, less harmful to the patients, especially those in advanced stages and/or individuals living with dementia.

Södersten et al., (2018) performed an analysis of histone modification patterns in neural progenitor cells and in differentiated dopaminergic and serotoninergic neurons [100]. They observed how the repression of progenitor genes and genes involved in the differentiation of each neuronal subtype is correlated with the distribution of epigenetic landscapes of gene activation and silencing for both adult neuron subtypes. Furthermore, they revealed a stress-induced differential gene expression profile in murine models of PD, which is characterized by the repression of various genes with a dual pattern of activation marks (H3K4me3 and H3K27me3) in promoter regions, especially in promoter regions of genes involved in cell cycle regulation. Toker et al., (2020), also suggested genome-wide alterations in histone acetylation profiles in PD-affected brains, triggering the hyperacetylation of H3K27 in genes such as *SNCA, PARK7*, *PRKN* and *MAPT*, which are associated with the development of PD [101].

Considering these studies, it is possible to infer that, although not fully understood, differential gene expression caused by alterations in chromatin states may be directly associated with the events involved in the pathogenesis of neurodegenerative disorders, such as the α-syn aggregation observed in PD. Moreover, it means that might the comprehensive map of epigenetic alterations in PD might be a good source of disease-specific biomarkers.

### 4.2. Non-Coding RNA Regulation

#### 4.2.1. MicroRNAs

MicroRNAs (miRNAs) are a class of 21-nucleotide long molecules, distributed in a wide range of organisms [102,103,104]. They are known for their role in post-translational gene silencing in which they are loaded into Argonaut proteins, forming RNA-induced silencing complexes (miRISC) to mediate the degradation of the target messenger RNA (mRNA). In human cells, most of the canonical miRNAs are transcribed from intronic sequences of coding and non-coding regions, although there are miRNAs encoded from exons [103]. Additionally, many miRNA loci are in close proximity, forming clusters, and miRNAs within the same cluster are usually co-transcribed, although some might be suppressed depending on cellular necessity [103].

Briefly, a mature miRNA binds to the argonaut 2 protein (AGO2), forming a complex called miRISC [102,103]. Here, the miRNA molecule works as a guide to recognition of the target mRNA, meanwhile the AGO2 works as an effector of the endonucleolytic cleavage of the mRy6 NA [102,105]. The recognition of the target mRNA depends on the complementarity between the miRNA binding site—located at the 3′ UTR region of the mRNA, and the seed region of the miRNA [102]. Depending on the strength of this complementarity, the mRNA can (i) be cleaved by the miRISC (in case of the perfect match); or (ii) the complex can mediate translational repression through the recruitment of additional proteins and enzymes that will degrade the mRNA by decapping and deadenylation [106].

This mechanism is part of a strictly regulated network and it was reported that, in neurons, the regulation of miRNAs was related to virtually all neuronal functions, including neurogenesis and neural development [107,108,109], synaptic plasticity [110,111], neural activities [112,113], and processes related to learning and memory [114]. Therefore, it would be expected that the deregulation of miRNAs would affect these processes, potentially leading to disease. In fact, age-related alterations in miRNA profile were reported in several studies [115,116].

Moreover, the deregulation of miRNAs was reported in several neurodevelopmental and neurodegenerative diseases, such as autism spectrum disorder [117], schizophrenia [118,119], and Huntington’s disease [120]. It also has been linked to the pathogenesis and progression of Alzheimer’s disease (AD) [121,122] and PD [123].

Several studies have demonstrated the potential of miRNAs for acting as PD biomarkers and therapeutic targets given their role in regulating genes classically associated with PD pathogenesis. Cardo et al., (2014) revealed a set of 11 miRNAs in the SNpc of PD patients [124]. Kim et al., (2007) reported that miR-133b was specifically expressed in midbrain dopaminergic neurons, associated with cell survival, and that PD patients presented hypoexpression of this miRNA [125]. MiR-7, miR-153 and miR-433 were shown to confer neuroprotective effects by regulating the levels of α-syn, and downregulation of these miRNAs was associated with *SNCA* overexpression and increased oxidative stress [126,127,128]. In PD models, Thome et al., (2016) [129] showed that miR-155 overexpression was associated with proinflammatory responses to α-syn aggregation. As α-syn aggregates are one of the various sources of oxidative stress in PD, these findings highlight the importance of further investigating the role of miRNAs in this disease.

Besides α-syn, it was demonstrated that miR-205 regulates the expression of *LRRK2* contributing to a pathogenic increase of this gene in PD patients [130] and Gehrke et al., (2010) demonstrated a bidirectional regulation between miRNAs and *LRRK2*, where pathogenic variants of the gene modulated the levels of miR-let-7 and miR-184 [131]. In addition, Xiong et al., (2014) [132] showed that miR-494 is directly involved in PD pathogenesis by targeting *DJ-1* and that the upregulation of the miRNA leads to hypoexpression of the gene. Furthermore, in a recent study, miR-221 levels were demonstrated to be modulated by the expression of *DJ-1*, a function lost in pathogenic mutants [133], and miR-221 showed a neuroprotective effect against the exposure to the dopaminergic neurotoxin 1-methyl-4-phenylpyridinium (MPP+) and the inhibition of the miRNA reduced the cell survival response against the MPP+-induced neurotoxicity.

It is important to emphasize that disease-induced deregulation of miRNAs (and some other ncRNAs) can be detected in patient blood circulation. Blood-based biomarkers are important for their applicability in clinical practice, for tracing of individuals at risk (especially among patient relatives), early diagnosis, and prognosis. Furthermore, in the case of neurodegenerative disorders, it is common to observe the overlapping of symptoms leading to additional complexity in identifying the disease [12,134]. Hence, circulating biomarkers may be helpful for differential diagnosis among neurological diseases, and the deregulation of ncRNAs such as miRNAs is a potential source of these biomarkers. For instance, mir-7 controls dopaminergic neuron and oligodendroglial cell fate by regulating genes of Wnt/β-catenin signaling [135]. Moreover, Zhang et al., (2019) observed that mir-let-7a can suppress neuroinflammation caused by microglia activation in PD patients [136]. Moreover, research of miRNA expression profiles identified apoptotic, inflammatory and axonal guidance pathways shared by PD and Alzheimer’s disease [137]. Another study revealed a unique molecular signature based on the deregulation of miRNAs and piRNAs in Alzheimer’s disease [121]. To our knowledge, this was the first study to investigate both small ncRNA classes in these diseases, and here we suggest that this approach could also be used to aid in PD diagnosis and patient management.

Recent studies have investigated the functional roles of miRNAs and other ncRNAs in neurodegeneration, exploring aspects such as the expression profile of miRNAs and potential target genes, thus indicating the biological processes affected by the deregulation of these molecules. One of the main points that remain to be elucidated is the identification of miRNA species that are shared among neurodegenerative disorders and disease-specific features miRNAs.

#### 4.2.2. Piwi-Interacting RNAs

PIWI-interacting RNAs (piRNAs) are molecules with up to 31 nucleotides in length that were identified in 2006, whose sequences are, generally, not conserved [138,139,140]. Currently, there are at least 30,000 piRNAs described in the human genome and they are suggested to be the most diverse class of small ncRNAs [141]. PiRNAs bind to proteins of the Argonaut/PIWI subfamily—in humans called Piwi-like (Piwil) proteins, forming regulatory complexes (piRISC) that perform (i) post-transcriptional gene silencing by mediating the deadenylation or endonucleolytic cleavages of the mRNA; and (ii) transcriptional silencing by inducing chromatin remodeling by interacting with proteins involved in this process [142,143,144].

They are highly abundant and mostly described in germline cells—especially in models such as *Drosophila melanogaster*, *Mus musculus*, and *Caenorhabditis elegans*. The first and main function attributed to piRNAs was the silencing of TE, which is the reason why they are known as the guardians of genome integrity [143,145]. However, not all piRNAs target TE sequences, indicating that piRNA regulation might be more complex than what was first thought [146]. Further studies in germline and somatic cells revealed other functions, including the euchromatin remodeling and epigenetic reprogramming [147,148], modulation of mRNA stability [140], and regulation of the translation of protein-encoding genes, sequences of other ncRNAs (such as long non-coding RNA—lncRNAs), and pseudogenes [149,150,151].

Currently, there are several reports concerning their role in the progression of several diseases, especially cancer. The deregulation of piRNAs was reported in various tumors such as gastric [2,152], colorectal [153], and breast [154] cancers, being associated with biological processes that contribute to tumor progression and metastasis. Meanwhile, their functions in the central nervous system (CNS) and their roles in the development of neurodegenerative diseases remain poorly understood. Among somatic cells, piRNAs are slightly more abundant in the brain [155,156]. Rouget et al., (2010) observed that impaired piRNA regulation of the mRNA encoding the posterior morphogen Nanos (Nos) in *D. melanogaster* early embryo leads to head development malformations [157]. Studies using MILI-piRNA mutant mice showed that piRNA pathways may act in locomotory and exploratory drives and normal anxiety-like behavior [86].

Notably, the mechanisms of piRNAs have been investigated in different organisms. For instance, Lee et al., (2011) reported the presence of piRNAs in murine brain and hippocampus cells, where they were associated with the regulation of genes related to the dendritic spine development and the organization of postsynaptic density [158]. In addition, Yan et al., (2011) reported the presence of piRNAs in neurons of Rhesus macaque (*Macaca mulatta*) [159]. Rajasethupathy et al., (2012) suggested that, in cells of the sea worm of genus *Aplysia*, neuron-specific piRNAs were associated with neuronal plasticity, learning, and the establishment of long-term memories; the synaptic release of the serotonin neurotransmitter induces the neurotransmitter-dependent activation of piRNAs and, in response, serotonin-induced piRNAs modulate DNA methylation patterns in the promoter region of the gene encoding the activating transcription factor 4 (CREB2) [151]. The CREB protein family is composed of transcription factors mediating cAMP responses, associated with neuroprotective pathways, related to neuronal plasticity, neurogenesis and memory formation [160,161]. CREB2 is also an antagonist of CREB1, which is modulated by miRNAs, and the modulation of CREB2 and CREB1 neurons may signalize which neurons hold memory traces and which are susceptible to draw new memories [151].

The interplay among piRNA and other ncRNAs was demonstrated to be important for regulating the permeability of the blood–brain barrier (BBB) in patients diagnosed with glioma. As revealed by Shen et al., (2018), the deregulation of the piR-DQ593109/Piwil1 complex leads to the upregulation of lncRNA maternally expressed 3 (MEG3)—which sponges the miRNA miR-330-5p [162]. Therefore, MEG3 upregulation leads to an increased hijacking of this miRNA. Meanwhile, miR-330-5p targets the Runt-related transcription factor (*RUNX3*), which binds to the promoter region of genes encoding the proteins occludin, claudin-5, and Zonula Occludens-1 (ZO-1), associated with the enhancement BBB permeability. Hence, the downregulation of miR-330-5p contributes to increased BBB permeability by promoting the upregulation of *RUNX3*, and consequently causing the hyperexpression of ZO-1, occludin and claudin-5 [162].

There is evidence linking piRNAs to the pathological features of multiple CNS-related diseases. For instance, piRNA deregulation was reported in studies involving Ischemia, Rett, Alzheimer’s disease, and PD [155,163,164,165,166]. Additionally, in neurons of *C. elegans*, the piRNA pathway was related to the inhibition of axonal regeneration via post-transcriptional regulation of the target genes, suggesting that understanding piRNA biology in the brain may be relevant to the treatment of neuronal injuries [167].

It is important to consider the roles of piRNAs in aging and in the progression of neurodegenerative diseases. Differentially expressed (DE) piRNAs play important roles in apoptotic cell death and neurodegeneration in Alzheimer’s Disease [164]. Alterations in the expression profile of piRNAs were also reported among cell lines of healthy and sporadic PD patients, as revealed by Schulzer et al., (2018) [163]. In this study, piRNA expression profile was able to distinguish cell lines of (i) fibroblasts; (ii) fibroblast-derived induced pluripotent stem cells (iPSCs); and (iii) midbrain neurons, from healthy individuals and PD patients. The authors revealed a PD-specific piRNA signature in all three cell lines, as well as the presence of Piwil2 and Piwil4 proteins in midbrain neurons. Among the downregulated piRNA set, there was an enrichment of Line-derived piRNAs, indicating the activation of these TEs. They also observed that CREB pathways were impaired in PD patients [163]. As discussed above, CREB pathways are involved in neuroprotective processes and some elements of this pathway are regulated by piRNAs in response to neurotransmitter activities [151,160,161]. Therefore, piRNAs might play an important role in PD development, although more studies are needed to further comprehend this process.

Moreover, recent studies have reported the occurrence of polymorphisms lying within piRNA genes, although the effects of these variants are still not well understood, especially when related to neurodegenerative diseases, since most studies are focused on cancer-related polymorphisms [155,164]. Single-Nucleotide Polymorphisms (SNPs) in piRNA genes were associated with increased risk for breast cancer [168], melanoma [169], and glioma [170] development, suggesting a probable impact of SNPs in piRNA functions in the CNS.

Finally, as mitochondrial dysfunctions are believed to play key roles in PD progression, the discovery of mitochondrial piRNAs in cancer cells [171,172,173] derived from genes involved in the oxidative phosphorylation chain, indicate a more complex regulatory network involving mitochondrial DNA (mtDNA), and is one more reason we should consider investigating the functional roles of piRNAs in healthy CNS and in disease onset.

#### 4.2.3. Long-Noncoding RNAs

Long non-coding RNAs (lncRNA) are the most studied class of ncRNAs. The term is commonly used to refer to linear RNA molecules with more than 200 nucleotides that may be involved in various processes such as DNA translation—acting as enhancers, protein scaffolds, guides for transcription factors, regulating pre-mRNA splicing and other processes [174,175]. Thus, it is suggested that lncRNAs can be involved in several mechanisms of disease onset, as reported in oncology studies. However, regarding PD, few studies associated the deregulation of lncRNAs with PD progression by regulating miRNA repression in neurons. Taurine Upregulated Gene 1 (*TUG1*) was associated with PD by regulating the miR-152-3p/PTEN pathway [176]. LncRNA GAS5 acts as a sponge of miR-223-3p, promoting microglial inflammatory response in PD by regulating inflammatory pathways [177].

LncRNAs also have a potential effect on PD onset and progression for being associated with damage and inflammatory responses in microglia and dopaminergic neurons. This is the case of lncRNA AK039862, for which upregulation was associated with the neuronal injury provoked by pesticides, inhibiting dopaminergic neuron proliferation and microglia migration [178]. NEAT1 is another lncRNA that is overexpressed in PD patients. Several studies demonstrated that its downregulation represses α-syn-induced activation of NLRP3 inflammation, apoptosis, and cytotoxicity by targeting miR-1301-3p, the miR-212-5p/RAB3IP axis, miR-124-3p and miR-212-3p [179,180,181]. The deregulation of NEAT1 downregulates microRNA-212-3p to accelerate the progression of PD. The knockdown of NEAT1 negatively affects the expression of *AXIN1*, a target of miR-212_3p reversing the suppression effect [179], meaning that the deregulation of this lncRNA is associated with PD development, characterizing it as a candidate therapeutic target. Furthermore, increased levels of NEAT1 were also detected in peripheral blood cells of patients with PD [182], revealing its potential as a PD progression biomarker.

Regarding lncRNAs in PD patient biofluids, the downregulation of lncRNAs MEG3 was reported as a potential PD biomarker in the plasma of PD patients when compared to that of healthy individuals, being associated with the aggravation of non-motor symptoms, cognitive decline, and PD stage [183]. Additionally, lncRNAs and α-syn were detected in plasma L1CAM exosomes and, combined with β-Glucocerebrosidase activity in plasma, they were correlated with the increase in motor/cognitive impairment [184].

In murine models of PD, lncRNAs decreased during PD development and were further decreased after the administration of L-DOPA therapy [185], suggesting that this set of lncRNAs were associated with both PD and Levodopa-induced dyskinesia (LID) pathogenesis, the latter is a common complication of the chronic dopamine replacement therapy. Gene Ontology analysis revealed the involvement of lncRNAs in processes such as oxidative stress response, inflammation, neurotransmission and apoptosis [185].

#### 4.2.4. Circular RNAs

Circular RNAs (circRNAs) are a class of long ncRNAs formed by the covalent bonding of their 5′-3′ ends. The lack of free terminations makes them resistant to degradation by exonucleases and, therefore, highly stable [186,187]. The biogenesis of circRNAs occurs in parallel with mRNA transcription in a process called back-splicing, processed with the spliceosome machinery, cis sequences in flanking introns, and some specific regulatory proteins [188,189].

About 183.000 circRNAs have been identified in humans [188,190,191]. They function by regulating gene expression mainly at the transcriptional and post-transcriptional levels, can act as microRNA or protein scaffolds and some can be translated [187,192]. Of all the known circRNAs, about 20% are produced by genes of neural function, making circRNAs significantly more abundant in nervous tissue compared to other tissues [187]. This can be explained, in part, by the high number of exons and long introns present in neural genes, making them more likely to be back-spliced [193]. In addition, studies report that the levels of circRNAs, produced in the CNS and other tissues are higher in the brain, suggesting specific neuronal regulation [193,194]. In the CNS, most circRNAs are produced by synaptic function genes and are transported from the cell body to the dendrites, indicating a potential role in neuronal communication [187]. As they are more stable than linear RNAs and extremely abundant in the CNS, it is suggested that circRNAs may serve as potential biomarkers of neurological disorders [195].

Some studies have demonstrated a relationship between changes in circRNA expression and the development of PD [187,196,197]. Among these circRNAs, ciRS-7, which stands out as the most abundant and the most studied in the CNS, is highly expressed in neurons. It has 73 binding sites for miR-7, working as a sponge for this microRNA that regulates several genes, including *SNCA* [187,197]. Cells transfected with miR-7 impair α-syn production, which is restored with the overexpression of ciRS-7 [128,197]. Another circRNA involved in the regulation of miR-7 is circSNCA. Sang et al., (2018) [196] demonstrated that the increased expression of this circRNA leads to increased production of α-syn.

Another circRNA, CircDLGAP4 has shown a key role in the PD pathogenesis, as its expression was significantly reduced and in 1-methyl-4-phenyl-1,2,5,6-tetrahydropyridine (MPTP+) cell cultures and in animal models with MPTP-induced PD. Among its functions, CircDLGAP4 can reduce mitochondrial damage and apoptosis, attenuate the neurotoxic effect of MPP+, and increase autophagy and cell viability [198]. In silico investigations suggested the regulation of miR-124-5p by the circDLGAP4 [198]. This miRNA directly targets genes involved in the CREB pathway, which is essential for the expression of neuroprotective factors, such as BDNF, BCL-2, and PGC-1α. Feng et al., (2019) demonstrated in MPTP-induced PD cellular and animal models that a reduction in circDLGAP4 expression leads to an increased expression of miR-134-5p. This suggests that the circDLGAP4/miR-134-5p/CREB axis has a key role in PD onset and progression [198].

Hanan et al., (2020) [199] stated that in PD brain tissues, the SNpc had a lower number of expressed circRNAs when compared to other brain regions. In contrast, circSLC8A1 was significantly more expressed in PD. They also observed that the increase in oxidative stress leads to higher levels of this circRNA. Despite this, there was no change in the expression of host mRNA SLC8A1, previously associated with neurodegeneration [199]. Data suggest that alterations of circSLC8A1 expression occur due to either increased circularization or deregulation and consequent reduced degradation [199,200]. CircSLC8A1 has seven miR-128 binding sites, participating in the regulation of this microRNA. After circSLC8A1 knockdown, 24 miR-128 target mRNAs were differentially expressed, including neurodegenerative regulators, dopaminergic neuron protectors, regulators of mitochondrial function, and mRNAs involved in chronic inflammation [201,202]. Recent studies have focused on evaluating the roles of circRNAs in CNS disorders. Despite that, few studies focus on the role of circRNAs in PD [193,194].

## 5. Mitochondrial Genetics and Epigenetics in Parkinson’s Disease

Mitochondria are cytoplasmic organelles that are involved in essential processes for proper cellular functioning, such as calcium (Ca^2+^) buffering, regulation of cell death, lipid homeostasis, among other metabolic signals [203,204], but their most notable function is the generation of chemical energy (ATP, adenosine triphosphate) through the electron transport chain (ETC) in the process of oxidative phosphorylation (OXPHOS) [173,204,205].

The mtDNA encodes 13 proteins for OXPHOS complexes and 24 encode for RNA molecules (22 tRNAs and two rRNAs), besides containing non-coding regions such as the displacement loop (D-loop), where the sequences for the initiation of replication and transcription are located [206]. Mitochondria also need 1200–1400 nuclei-encoded proteins for their overall functioning, a process controlled by mitonuclear communication, which is crucial for the performance of both parties and the consequent cellular balance, including energy generation [207,208]. For instance, the ETC comprises five protein complexes (I-V), of which four (complexes I, III, IV and V) are encoded by both the mitochondrial genome (mtGenome) and the nuclear genome, and one (complex II) is encoded exclusively by the nuclear genome [205,209].

Remarkably, most ATP is produced during OXPHOS and, thus, mitochondrial dysfunctions would greatly affect the energy supply. These dysfunctions may be caused by genetic or epigenetic alterations. In this sense, it should be noted that reactive oxygen species (ROS) are also generated during OXPHOS; in excess, ROS may lead to cellular damages and oxidative stress, with a special impact on mitochondria. Therefore, mtDNA is particularly vulnerable to oxidative damage and mutations, which in turn may affect mitochondrial bioenergetics and increase ROS production [203,210]. Considering that neurons demand high ATP rates, mitochondrial malfunction might have a particular influence on the energy generation of these cells, impairing neural circuitry homeostasis, neurotransmission and neuroplasticity [211,212,213].

Mitochondria started to be associated with the etiology of PD from the observation of animal models developing parkinsonism after treatment with (MPTP) [214] and, in the following years, with other drugs such as rotenone, all potent neurotoxins that are now known to inhibit complex I activities in the SNpc [215]. Indeed, defects in complex I were identified in different tissues of PD patients, including the SN of postmortem brains [216], skeletal muscles [217] and platelets [218]. Interestingly, the transfection of platelet mtDNA from PD patients into normal cell lines caused the reduction of complex I and IV activity in the receiving cells [219]. While malfunction of Complex I seems to have an important role in the increase of neurotoxic vulnerability, oxidative stress and consequent loss of dopaminergic neurons, other mitochondrial mechanisms have also been pointed out as PD-related factors, such as calcium regulation, biogenesis, dynamics and mitophagy [220].

Currently, there is an increasing interest in the possibility of mtDNA variants predisposing to idiopathic PD [221,222,223]. Several studies have suggested an association of SNPs in mitochondrial genes with an enhanced risk for PD, such as 4216T>C in *MT-ND1* [224], 5460G>A in *MT-ND2*, as well as 4336T>C [225,226] and 4336A>G in *MT-TQ* [227]. Whole mtGenome sequencing of PD patients revealed an accumulation of mtDNA deletions in SN dopaminergic neurons [228] and heteroplasmic variants in genes of complex III (*CYTB*) and complex IV (*COXI* and *COXII*) in SN and frontal cortex tissues [229].

Although defects in complex I have been extensively shown as important etiological factors for idiopathic PD, the sequencing of whole mtGenome [228,229] or specific complex I genes [226] has failed to find variants that explain such defects and only a few studies have found mitochondrial SNPs associated with the risk of PD [224,230]. Interestingly, in different studies, the variants 10398A>G (*MT-ND3*) [231], 2158T>C (*MT-RNR2*) and 11251A>G (*MT-ND4*) [232], related to the classification of some European mitochondrial haplogroups, were associated with reduced risk to PD. Considering these studies together, haplogroups JT, T, J or K seem to present a decrease in the risk of PD in comparison to other European haplogroups (H or HV).

Dysfunctional mitochondria are also related to monogenic forms of PD: genetic methods have identified variants in mitophagy regulatory genes—e.g., *PINK1* (PTEN-induced putative protein kinase 1), *Parkin* (*PRKN*, parkin RBR E3 ubiquitin protein ligase) and *DJ-1* (protein deglycase DJ-1)—as hereditary factors of PD etiology, being associated with early-onset autosomal recessive PD (age less than 45 years) through alterations in mitophagy [233,234]. In order to maintain mitochondrial homeostasis in the nervous system, mitophagy (the process of autophagic degradation of damaged mitochondria) may take place, mainly through the PINK1/Parkin pathway [223,235]. Thus, considering mitophagy is part of mitochondrial quality control, variants in genes related to this mechanism may impair the elimination of dysfunctional mitochondria in the brain of PD patients [236].

Over 20 years ago, the *Parkin* gene was first described and associated with autosomal recessive juvenile parkinsonism in Japanese families (being named *PARK2*) [237]. A few years later, independent studies have mapped novel loci and associated them with early-onset PD: one in an Italian family [238] and the other in the Netherlands [239], being named *PARK6* and *PARK7*, which were later related to *PINK1* and *DJ-1* genes, respectively. Since then, many studies have suggested the association of genetic factors in mitophagy with PD. Recently, a review by Cai and Jeong (2020) [240] highlighted that defects in mitophagy have been widely associated with PD (especially in the PINK1/Parkin pathway but also in others like lipid-mediated mitophagy) and that increased rates of mtDNA deletions have been observed in PD patients, which could be related to dysfunctions in this process of mitochondrial quality control.

In addition to mtDNA variants, epigenetic mechanisms such as DNA methylation and the presence of ncRNAs have also been described in these organelles, giving birth to a research field that is now called mitochondrial epigenetics (mitoepigenetics) [173]. Since the 1970s, the occurrence of mtDNA methylation has been widely discussed and considered to be controversial; currently, not only is this type of epigenetic regulation recognized in mitochondria, but it is known that the metabolism of these organelles is involved in regulating the production of the universal methyl donor (S-adenosylmethionine, SAM) [241,242]. In addition, a study investigating mtDNA methylation in different brain tissues demonstrated that such patterns in the mitochondrial epigenome may vary among tissues [243]. Recently, a study reported genome-wide mtDNA methylation in different cell lines and tissue samples, providing patterns in both CpG and non-CpG contexts (but especially in non-CpG sites) and suggesting the involvement of varied DNA-methyltransferases (DNMT1, DNMT3A and DNMT3B) in this mitochondrial process [244].

As for ncRNAs, their discovery in mitochondria is more recent than mtDNA methylation. In 2011, a study reported the first human mitochondrial transcriptome and reinforced some ncRNAs shown in mitochondria a few years earlier, highlighting novel levels of complexity in mitochondrial regulation and encouraging the investigation of ncRNAs in these organelles [245]. Since then, different classes of ncRNAs have been described in human mitochondria (circRNAs, miRNAs, piRNAs, lncRNAs and sncRNAs), as recently reviewed by Cavalcante et al., (2020) [173]. These ncRNAs in mitochondria are known to participate in mitonuclear communication and may be either nuclear-encoded ncRNAs (nuclear-ncRNAs) or mitochondrial-encoded ncRNAs (mt-ncRNAs), involved in anterograde signaling (nucleus regulating mitochondria) or retrograde signaling (mitochondria regulating nucleus), respectively. Although many aspects of these mechanisms remain largely unknown, several mitochondrial-located lncRNAs and miRNAs have presented target genes involved in different mitochondrial functions [246].

In this context, epigenetic mechanisms in mitochondria have been associated with neurodegenerative diseases such as PD. For instance, reduced levels of mtDNA methylation (5-methylcytosine, 5mC) were observed in the D-loop region (but not in the other analyzed region, *MT-ND6*, which encodes a subunit of Complex I) in the SNpc of PD patients when compared to controls [247]. It should be noted that the differential methylation in nuclear genes related to mitochondrial apoptosis in association with cognitive and motor progression in PD was also reported, reinforcing mitochondrial influence on the evolution of this disease [248]. Similarly, a recent review by Lyu et al., (2019) [249] highlighted that mitochondrial-related lncRNAs might also play a role in PD through mitochondrial dysfunction or abnormalities in processes related to the management of oxidative stress, like mitophagy and apoptosis. Although there are currently few studies on this matter, it is increasingly clear that mitochondrial epigenetics may play an important role in the development and progression of PD and should be given more attention. Therefore, the investigation of different aspects in both mitochondrial genetics and epigenetics is presented as a promising strategy in the search for biomarkers of onset and treatment of PD, known to be a multifactorial disease.

## 6. Gut Microbiome in Parkinson’s Disease

The gut microbiome (GM) consists of trillions of microorganisms that inhabit the human gastrointestinal tract and constantly interact with the host at multiple genetic and metabolic levels [250]. This community is known to have a strong effect on human health and its disruption has been associated with the development of various human diseases, such as inflammatory bowel disease, metabolic, immune, and neurodegenerative disorders [251,252]. The crosstalk between the microbiome, the gut and the nervous system is collectively referred to as the microbiota–gut–brain axis [253].

In Braak and colleagues’ dual-hit hypothesis regarding the staging of LB accumulation in neuronal cells [254], the enteric nervous system (ENS) is one of the sites in which neurodegeneration begins. In this scenario, α-synuclein toxic forms would initially invade the ENS and eventually reach the CNS via the vagus nerve, triggering PD motor symptoms. Several lines of evidence show support of such a mechanism. First, results show that complete vagotomy is effective in decreasing the risk of developing PD [255]. Secondly, chronic constipation is one of the major and widespread early symptoms of PD, affecting approximately 80% of patients and with such signals being detected decades before diagnosis [10]. Additionally, Sampson et al., (2016) [256] showed worsening of motor impairments when α-syn overexpressing mice were inoculated with the GM of PD patients when compared to non-PD control GM. Recently, such findings were corroborated with evidence showing exacerbated motor impairments in a mouse model of PD with gut dysbiosis and intestinal inflammation [257,258,259,260]. Altogether, these findings establish convincing evidence for the role of the GM in regulating motor deficits in PD development.

To date, no single microbial species or taxa has been determined to have a causal role in PD development. However, structural differences between the GM of PD patients and healthy controls have been previously documented and accumulating evidence shows the abundance of certain fecal microbial taxa are differentially distributed among PD patients and healthy controls. *Lactobacillus*, *Bifidobacterium*, *Verrucomicrobiaceae,* and *Akkermansia* show an increase in PD patients while *Faecalibacterium*, *Roseburia*, *Coprococcus*, *Blautia*, *Prevotella*, and *Prevotellaceae* have lower abundances [261,262,263,264].

Scheperjans et al., (2015) [265] found that the relative abundance of *Prevotellaceae* taxa was reduced by nearly 80% in PD patients when compared to controls, and that the abundance of four microbial taxa was able to classify PD patients with over 90% specificity. Further, disease phenotypes such as postural instability were associated with the abundance of *Enterobacteriaceae* in PD patients from the same cohort. More recently, Aho et al., (2019) also found that *Prevotella* taxa are less abundant in faster-progressing patients [261]. Hegelmaier et al., (2020) tested dietary and enema interventions in PD patients and observed changes in the abundance of certain bacterial taxa, such as *Ruminococcaceae* associated with improved motor symptoms and decreased levodopa daily doses [266]. Hertel et al., (2019) [267] and Baldini et al., (2020) [268] proposed that *B. wadsworthia*, found in higher abundances in PD patients, is crucial for sulfite production in the gut, thus mediating brain mitochondrial energy balance, acting as a neurotoxin and promoting impaired metabolite secretion in PD [267,268].

In fact, communication between the host and the gut microbiome often consists of interactions involving short-chain fatty acids (SCFA), a major group of metabolites produced by the GM from dietary substrates [269]. SCFA production is crucial to metabolic homeostasis promotes overall systemic health. For instance, butyrate and propionate, two of the main SCFAs, have neuroprotective effects and aid in the rescue of motor capacity in PD [270], However, fecal SCFA levels have been found to be reduced in PD patients [263,271]. Cirstea et al., (2020) [272] demonstrated that butyrate synthesis is reduced in PD while deleterious aminoacid metabolites are increased, aggravating gut inflammation and constipation [273]. GM is also responsible for converting dietary flavonols into phenolic acids, another important metabolite [273]. Ho et al., (2019) demonstrated that individual GM metabolic repertoires of polyphenol production have the ability to protect against neurological disorders involving α-syn toxicity [274].

The mechanisms by which the GM affects neurodegenerative conditions are likely impaired production of neuroprotective factors, increased levels of pro-inflammatory cytokines, and unbalanced immune responses [275]. A reduction in the abundance of anti-inflammatory and neuroprotective metabolite-producing bacteria, such as members of the *Lachnospiraceae* family, has been documented for PD patients [276]. Further, taxa such as Bacteroides and *Verrucomicrobia* species were found in positive correlation with the abundance of pro-inflammatory cytokines TNF-α and IFN-γ in Asian PD patients [264]. Another inflammation-mediated GM effect is through small intestinal bacterial overgrowth (SIBO), present in around 25% of PD patients [277,278]. SIBO, which influences gastrointestinal dysfunction, seems to interfere in PD pathogenesis by increasing intestinal permeability, pro-inflammatory cytokine activation and, consequently, microglial activation. This leads to worsening of motor capacities and may also interfere in levodopa absorption [278].

Host–microbiome interactions in PD gain another level of complexity when considering host-derived epigenetic interference upon microbial metabolism. It has been shown that gut microbial DNA expression is affected by ncRNA molecules produced by gut epithelial cells, and may influence important bacterial pathways [279]. Such findings still require validation and more fecal ncRNA data for PD are needed. However, such associations attest to the intricate involvement of the GM in PD pathogenesis and encourage future studies to investigate this relationship further.

Current therapeutic interventions related to the GM are focused on probiotics, prebiotics, antibiotics and fecal microbiota transplantations (FMT) in a wide range of disorders, such as cancer and neurological diseases [280,281]. Probiotics could be employed to improve gut health, and antibiotics have been shown to ameliorate motor dysfunction by preventing dopamine neuron loss [282,283]. To date, one report has shown a successful case of FMT in improving constipation and leg tremors in a PD patient [284]. In Alzheimer’s disease, FMT has also been shown to be useful for improving symptoms following *C. difficile* infections [285]. It is important to note that GM-based therapeutic approaches do not necessarily involve proof of the causal role of microbial communities in PD pathogenesis, considering the GM modulates various metabolic systems and could improve quality of life in diverse aspects, such as gastrointestinal inflammation or constipation [286].

Gut microbiome data may be employed to determine the initial steps and progression of PD, considering not all patients experience enteric neurodegeneration at first. Additionally, profiling and measurement of GM metabolites could be applied to the clinical environment once we determine its predictive value to disease progression and/or prognosis. This could lead to personalized approaches in patient management as the GM could become a crucial part of PD treatment and diagnosis [286].

## 7. Future Perspectives in Parkinson’s Disease

The multifactorial nature of PD poses a difficult obstacle for understanding the mechanisms involved in its onset and progression. However, it does provide a multitude of possibilities when it comes to the discovery of cellular pathways and biomarkers that can be employed for diagnostic or treatment purposes. Future efforts should focus on expanding population representation in PD databases as well as standardizing methods to avoid conflicting results due to different approaches.

Perspectives for PD biomarker discovery are promising as we invest in multi-omics techniques for characterizing patient genomic, epigenomic and gut microbial profiles. In this context, we might be moving towards personalized medicine approaches that employ a combination of different biomarkers. For instance, as GI tract alterations are one of the first symptoms related to PD onset, metagenomic data may be an important tool for early diagnosis.

Furthermore, the study of PD-related variants at a population level may also highlight individuals at risk, leading to early clinical interventions, and the role of ncRNAs may shed light on PD regulation mechanisms. Although there is no cure for PD yet, it is important to emphasize that the molecular factors involved in PD onset and progression may not only be applied for population screening and early diagnosis, they can also become new therapy targets, being involved in the development of new pharmacological approaches. Therefore, these potential biomarkers might help the development of innovative therapeutic approaches, elucidate neurodegeneration processes and improve patient care.

We would like to highlight that, considering the increasing amount of data from genomics, epigenomics, metagenomics, and other various omics, it is essential to analyze data in an integrative approach to understand the complexity of PD, find disease-specific biomarkers for population screening, early detection, as well as discovering new therapies to attenuate disease progress.

## 8. Conclusions

The post-genomic era has expanded our knowledge on several molecular processes, producing data that have been applied for early diagnosis and improvement of patient care in various diseases, such as cancer. However, the same is not a reality when it comes to neurodegenerative disorders, mainly due to scarce data and the obstacles in fully understanding the molecular mechanisms leading to neuronal death. The data discussed in this review highlight that genomic variation, regulation by epigenomic mechanisms, as well as the influence of host gut microbiome have a crucial role in the onset and progress of PD. The investigation of these molecular aspects in a collaborative and multidisciplinary manner is key to the rise of personalized medicine for PD patients.

## Figures and Tables

**Table 1:** Parkinson’s disease-associated genes.

Gene	Protein	PD Type	Main Function	Reference
*SNCA*	α-Synuclein	Monogenic and Sporadic PD	Increase local Ca^2+^ release to enhance ATP-induced exocytosis; Regulation of synaptic vesicle trafficking and neurotransmitter release; Modulation of Dopamine transporter (DAT)	[26] [33] [34] [31]
*GBA*	Glucocerebrosidase	Sporadic PD	Degradation of complex lipids; Cholesterol metabolism	[35] [36] [37] [38] [39]
*LRRK2*	Dardarin	Monogenic and Sporadic PD	Cellular response to Dopamine Mitochondrial organization, location Regulation of autophagy Mitochondrial depolarization	[40] [41] [42] [43] [44]
*PARK2*	Parkin	Familial and Sporadic PD	Mediates ubiquitination to remove and/or detox abnormal folded or damaged proteins	[45] [46]
*PARK6*	Pink1	Familial and Sporadic PD	Regulation of damaged mitochondrial clearance by mitophagy	[47] [45] [48]
*PARK7*	DJ-1	Sporadic PD	Mitophagy Response to ROS Regulation of neural apoptosis	[49] [50] [51] [52]
*MAPT*	TAU	Sporadic PD	Astrocyte activation; Axonal transport of mitochondria Microglial cellular activation Cellular response against ROS Regulation of Autophagy Regulation of Synaptic Plasticity	[53] [54] [55] [56]
